# Supplementary material for: Patterns of Intron Gain and Loss in Fungi
Source: PLoS Biol. 2004 Nov 30;2(12):e422. doi: 10.1371/journal.pbio.0020422 (PMC532390; doi:10.1371/journal.pbio.0020422)
Supplement: Table S1 — Also available at http://genes.mit.edu/NielsenEtAl/. (4.3 MB ZIP). [file pbio.0020422.st001.zip › NielsenEtAl/html/922.html]

AN3634.1.NCU04411.1.MG05168.1.FG00598.1


```
 CLUSTAL W (1.82) Multiple Sequence Alignments - Introns Inserted


Sequence 1: MG05168.1	425 aa
Sequence 2: FG00598.1	411 aa
Sequence 3: NCU04411.1	436 aa
Sequence 4: AN3634.1	426 aa
Alignment Length: 454 aa
Number Identitical Residues: 180 aa
Alignment Score (without introns) 9176


MG05168.1 	------MVAPNANGNGTELPKVDIKPLLSRLWPVPKEDINQVSVDDIADAIAHFFTDRVS
NCU04411.1	MSAQQQQAAPATATAAEDLPDVNIKKLLTRLWPLGHP--DPVTPDEIAHAISYFFTNQIS
FG00598.1 	------MAPQSQDSGQNALPPVDIKPLLTKLWPAES----AVSPQEIAEAISHFFTNQVT
AN3634.1  	----------MSASPRQKPESISISPLLKKLAYPASEL--AVEASEIASAFALIFEDRLS
          	                     :.*. **.:*          *  .:** *:: :* ::::

MG05168.1 	EVQAASLLMCLHFTGFDRRADVMAATAARMRSAAEKIDLTEINRIILDRNLGEGNYQGGL
NCU04411.1	DVQAGALLMCLHFTGLDRSAEVLSKTAQVMLKAAAQTDLNELRQVIEKRGKQEGAYNGGL
FG00598.1 	EAQTASLLMSLHFTKLDFRADVLAECARVMREAAAPIPVEELREVIERRGRKEGAYRGGL
AN3634.1  	DIQTAALLTLLHSTQLDKDAEVIAKCSHRMREAARQTDKAALRQAIQSRGRKEGNYRGGL
          	: *:.:**  ** * :*  *:*::  :  * .**       :.. *  *.  ** *.***

MG05168.1 	~VDIVGTGGDAHNTFNISTTSSIIASSLLMLAKHGNRSSTSKSGSADLLANMKPMPPNMD
NCU04411.1	0CDIVGTGGDSHNTFNISTTSSILASSLLLVGKHGNKASTSKSGSADLLACVPPRPPQIS
FG00598.1 	0CDIVGTGGDGHDTFNISTTSSILASALLMVSKHGNKASTSKSGSADLVACMKPQPPIIS
AN3634.1  	0CDIVGTGGDSHSTFNISTTSSILASPLLMMAKHGNRAQTSFSGSADVLNAITPVPPKIG
          	  ********.*.**********:**.**::.****::.** *****::  : * ** :.

MG05168.1 	SVAPTTLSKVYSETNYAFLFAPVFHTGMRYVAPIRKQLPWRTLFNLIGPLSNPIDSGV--
NCU04411.1	RIRPDTVCSLYSQCNYGFLFAPVFHQGMRHVAPIRAQLPWRTIFNLVGPLANPLDALDPA
FG00598.1 	AVRPDTLVKAYSETNYTFLFAPVFHTGMRYVAPIRKQLPWRTVFNNLGPLANPVED----
AN3634.1  	AVTAENLSQVYAASNYAFLFAPNFHYGMMYADNVRRNLGLRTIFNLMGPLANPVDW----
          	 : . .: . *:  ** ***** ** ** :.  :* :*  **:** :***:**::     

MG05168.1 	-------GSPVLEARVLGVARAELGPVFLDALRMAGVDKAMVICGVEELDEVSCAGPTNV
NCU04411.1	VFTGEGGKDKLVEARMIGCARKDLGPVFAEALKMGGARKAMVVCGDEELDEISCAGPTHC
FG00598.1 	----------VLEARVIGVGRRDLGPAFAEALCMAGFKKALIICGEEDLDEVSCAGNTLC
AN3634.1  	----------AIEARVVGVAYKALGPVFVEALRQNGVTKGLVICGEEDLDEISCAGKTNC
          	           :***::* .   ***.* :**   *  *.:::** *:***:**** *  

MG05168.1 	WRLRRR---------RDGEGNGE-----AVISEHFYVNPSDFGLSTHPLANVSGNKEPAE
NCU04411.1	WTIKPT---------EEDPEG-------EPVVDHFLLHPSDFGLPAHSLSEVSPGKEPIE
FG00598.1 	WKVNET---------SPGK----------LEVEHFTVHPSDFGLSTHPLNTVSSGKEPSE
AN3634.1  	WQLSEIPNPDYDLSKEDDEDEEYRTPPTIAKLDEFQLEPADFGLPSYPLTAVYGRKMPKD
          	* :   .... . :.  .      :..:    :.* :.*:****.::.*  *   * * :

MG05168.1 	NAEILSKILQGELPDDDPILEFVLMNTATLFVASGICEADQSNMGHGDDGVVIKERGPGG
NCU04411.1	NAAILSKILNNEMPDDDPILHFVLMNTAALFVVSGICEADTSDMGHGDDGQVVKERGPGG
FG00598.1 	NAEILSRILHNELPDNDPILEFVLLNTAALLVTSGICEADTSNMGKGDDGKVITERGPGG
AN3634.1  	NAAKLMQILRNELPRDDPILSFVLMNVAALLVTSGICEADSSNMGPGDDGQVITERGPGG
          	**  * :**..*:* :**** ***:*.*:*:*.******* *:** **** *:.******

MG05168.1 	ERWKEGVRRARWALKSGEAWRQWQAFVKVTNSISR
NCU04411.1	GRWKEGIRRARWAIKSGEAARQWAKFVEVTNSFSA
FG00598.1 	LRWKEGVRRARWALKSGEAWRQWEKFVKVTNEIGA
AN3634.1  	GRWKEGVRRARWAIESGSALKCFEEFIEVTNKL--
          	 *****:******::**.* : :  *::***.:
```
